# Clinical implantation of 92 VACStents in the upper gastrointestinal tract of 50 patients—applicability and safety analysis of an innovative endoscopic concept

**DOI:** 10.3389/fsurg.2023.1182094

**Published:** 2023-05-05

**Authors:** J. Lange, J. Knievel, D. Wichmann, G. Kähler, F. Wiedbrauck, T. Hellmich, M. Kandler, J. Bernhardt, D. Scholz, T. Beyna, J. Hausmann, E. Wedi, M. Ellrichmann, U. Hügle, A. J. Dormann, C. F. Eisenberger, M. M. Heiss

**Affiliations:** ^1^ Department of Abdominal, Tumor, Transplant and Vascular Surgery, Cologne-Merheim Medical Center, Witten/Herdecke University, Cologne, Germany; ^2^ Department for Visceral, General and Transplant Surgery, Tübingen University Hospital, Tübingen, Germany; ^3^ Multispecialty Endoscopy Center, Mannheim Medical Center, University of Heidelberg, Mannheim, Germany; ^4^ Department of Gastroenterology, AKH Celle, Celle, Germany; ^5^ Department of Gastroenterology, Städtisches Klinikum Dresden, Dresden, Germany; ^6^ Department of Surgery, Klinikum Suedstadt Rostock, Rostock, Germany; ^7^ Department of Gastroenterology and Metabolism, Ameos Klinikum Am Bürgerpark, Bremerhaven, Germany; ^8^ Department of General Internal Medicine and Gastroenterology, Evangelisches Krankenhaus Düsseldorf, Düsseldorf, Germany; ^9^ Department of Gastroenterology/Internal Medicine, St. Vinzenz-Hospital Hanau, Hanau, Germany; ^10^ Division of Gastroenterology, Gastrointestinal Oncology and Interventional Endoscopy, Sana Clinic Offenbach, Offenbach, Germany; ^11^ Department of Interdisciplinary Endoscopy, Medical Department 1, University Hospital Schleswig-Holstein, Campus Kiel, Kiel, Germany; ^12^ Department of Gastroenterology, Cologne-Holweide and Merheim Medical Center, Cologne, Germany

**Keywords:** endoscopic treatment by vacuum therapy (EVT), upper gastrointestinal wall defects, anastomotic leakage (AL) after esophagectomy, VACStent, preemptive therapy

## Abstract

**Introduction:**

Endoscopic vacuum therapy (EVT) has emerged as a promising treatment option for upper gastrointestinal wall defects, offering benefits such as evacuation of secretions and removal of wound debris by suction, and reduction and healing of wound cavities to improve clinical outcomes. In contrast, covered stents have a high rate of migration and lack functional drainage, while endoluminal EVT devices obstruct the GI tract. The VACStent is a novel device that combines the benefits of EVT and stent placement. Its design features a fully covered Nitinol-stent within a polyurethane sponge cylinder, enabling EVT while maintaining stent patency.

**Methods:**

This study analyzes the pooled data from three different prospective study cohorts to assess the safe practicality of VACStent placement, complete leak coverage, and effective suction-treatment of esophageal leaks. By pooling the data, the study aims to provide a broader base for analysis.

**Results:**

In total, trans-nasal derivation of the catheter, suction and drainage of secretion via vacuum pump were performed without any adversity. In the pooled study cohort of 92 VACStent applications, the mean stent indwelling time was 5.2 days (range 2–8 days) without any dislocation of the device. Removal of the VACStent was done without complication, in one case the sponge was lost but subsequently fully preserved. Minor local erosions and bleeding and one subsequent hemostasis were recorded unfrequently during withdrawal of the device (5.4%, 5/92) but no perforation or pressure ulcer. Despite a high heterogeneity regarding primary disease and pretreatments a cure rate of 76% (38/50 patients) could be achieved.

**Discussion:**

In summary, insertion and release procedure was regarded as easy and simple with a low potential of dislocation. The VACStent was well tolerated by the patient while keeping the drainage function of the sponge achieving directly a wound closure by continuous suction and improving the healing process. The implantation of the VACStent provides a promising new procedure for improved clinical treatment in various indications of the upper gastrointestinal wall, which should be validated in larger clinical studies.

**Clinical Trial Registration:** Identifier [DRKS00016048 and NCT04884334].

## Introduction

Originally developed by surgical endoscopists for the treatment of anastomotic leakage (AL) in the lower gastrointestinal (GI) tract after rectal surgery, endoluminal vacuum therapy (EVT) has also been used in the esophagus and upper GI tract (1).

EVT involves the use of a suction pump and airtight sealing technique to create a closed environment and apply negative pressure to the wound compartment. In comparison to endoscopic stenting, several retrospective studies have shown that EVT has a higher success rate in closing AL, a shorter treatment duration, and a lower mortality rate (2, 3).

Indications for using EVT in the upper gastrointestinal tract include the treatment or prevention of suture line leaks after resections of the upper GI tract and bariatric procedures, iatrogenic perforations, transmural wall defects of the esophagus and the esophagogastric junction, and spontaneous ruptures (Boerhaave syndrome) (4).

The use of covered stents in endoluminal vacuum therapy (EVT) is limited due to high migration rates and the lack of functional drainage (5). To address these issues, a new device called the VACStent has been developed, which combines the benefits of EVT and covered stents. The VACStent is a self-expanding nitinol stent covered with a silicone membrane and encased in a polyurethane sponge cylinder. A nasal suction catheter is embedded in the open-cell PU sponge and connected to an adjustable vacuum pump. The ends of the covered stent are in contact with the intestinal wall, sealing it from the lumen. Negative pressure is created in the area of the sponge cylinder by the suction catheter, which allows for effective drainage and generates an excellent fixation of the VACStent to the intestinal wall.

Initial clinical applications of the VACStent have shown that it is safe and effective, and that the innovative design-related features of the device have clinical benefits (6, 7).

The aim of this pooled analysis of three different patient cohorts was to evaluate the clinical handling of the VACStent and the technical application in a larger patient population. This offers the opportunity to analyze with a broader database for complications and get a better quantitative judgement of safety and applicability.

## Method and materials

The VACStent (VacStent GI™) is a medical device developed by VACStent GmbH in Fulda, Germany and distributed by MICRO-TECH Europe GmbH, Duesseldorf, Germany. It is classified as a Class IIa medical device according to European conformity certification (CE). The VACStent is composed of a fully covered intestinal stent, a polyurethane sponge cylinder, and a suction catheter within the sponge that is connected to a vacuum pump. The stent is securely attached by suction to the gastrointestinal wall, which prevents migration and slippage while allowing for the passage of nutrition and liquid during ongoing endoluminal vacuum therapy (7). The VACStent is loaded into an introducer-system and can be applied endoscopically over-the-wire. Once in place, the device can be released and expanded to an appropriate shape. The flanged ends of the VACStent frame the sponge cylinder against the intestinal lumen allowing for circular vacuum therapy over the full length of the sponge cylinder**.**

## VACStent application

During transoral endoscopy a stiff guide wire was placed under direct vision in the gastric-tube or duodenum. The delivery system was then carefully advanced over the wire and the VACStent deployment was observed via a small 8 mm endoscope that runs parallel to the delivery system. Once the VACStent was in place, the application system and guide wire were removed and the suction catheter was passed retrograde through the nose. Before connecting the suction catheter to a VAC-pump (e.g., Curasul®, BSN medical GmbH, Hamburg, Germany) using a plastic Y-adapter, the sponge cylinder was rinsed retrograde with 0.9% NaCl solution to facilitate and ensure the deployment of the open-cell polyurethane sponge. In all cases the continuous suction pressure was −75 to −125 mmHg.

Before removing the VACStent, it is recommended to extensively rinse the sponge retrograde via the drainage-tube with at least 40 ml 0.9% NaCl solution. Additionally, suction should be stopped for at least 2 h before VACStent removal. Endoscopic forceps were used to pull at the retrieval loops placed at the ends of the VACStent to withdraw the device.

The recommended retention time for a VACStent was 3 to 7 days, in case of anastomotic leakage with an expected average number of 3 stents for successful closure.

## Patients

Three study cohorts were analyzed:

The first cohort entails the patients of an already published prospective multicenter open-label study where 41 VACStents had been implanted in 15 patients with different, mostly anastomotic leaks (8). Experienced endoscopists conducted the study at three tertiary centers in Germany, namely Klinikum Südstadt Rostock, Mannheim Medical Center, and Cologne Merheim Medical Center. The study was approved by the Institutional Review Board (IRB) of Witten/Herdecke University (No. 124/2018) and other relevant IRBs. The trial was registered with the German Registry of Clinical Trials (DRKS00016048). Patients who were diagnosed with an upper gastrointestinal (GI) leak, either postoperatively at an anastomotic suture line or as a result of an iatrogenic leak caused by an endoscopic (e.g., TEE) or surgical procedure, were included in the study. The diagnosis was confirmed through endoscopy. Patients with leaks not endoscopically accessible with the VACStent, clinically unstable septic patients, with severe permanent vomiting, with clinical ileus signs, or a need for full anticoagulation or with thrombocytopenia <20.000/µl were excluded.

Two further cohorts from an open-label multicenter register study were included in this analysis (Study centers Celle, Kiel, Bremerhaven, Dresden, Düsseldorf, Hanau, Tübingen, Offenbach, Cologne). The study protocol was approved of the Institutional Review Board of the Witten/Herdecke University (No. 34/2020,), VAC-Stent registry: NCT04884334. One cohort entails 25 patients with 39 implantations for esophageal leakages. Eligible was any patient with endoscopic confirmation of a diagnosis of upper GI leak (postoperatively, iatrogenic, spontaneously).

A second cohort comprises 12 VACStent implantations in 10 patients in a preemptive setting receiving the VACStent during esophageal resections. All of these patients have been enrolled at the Cologne-Merheim Medical Center and all patients have a history of chemotherapy or radiochemotherapy for esophageal cancer (9).

Participation in these studies was based solely on a written consent form that was signed by all patients.

## Data collection and analysis

The safety, efficacy, and clinical progress of VACStent treatment were monitored on a daily basis, starting from patient enrollment until hospital discharge, and during follow-up visits up to 12 months post-operation. Long-term data on the VACStent treatment were obtained through follow-up endoscopy. All data were collected in a case report form (CRF), entered into a database, and analyzed.

## Analysis endpoints

The primary endpoints of the analysis were complete coverage of the leak, safe feasibility, and effective suction-treatment of esophageal leaks or the esophageal-gastric anastomoses in the preemptive setting. The secondary endpoints included successful healing of the leak, prevention of septic conditions, and the incidence of complications, specifically bleeding, stent migration, and local erosions.

## Results

In total, 50 patients received 92 VACStents as part of two clinical trials in Germany between August 2019 and January 2023 (Table 1). The patients were aged from 23 to 92 and had an ASA Score from 1 to 4, most of them ASA 3 (64%).

**Table 1:** Patients and procedures.

Patient characteristics (*n* = 50)	
Patients with esophageal leakage, *n*	40
Patients with preemptive VACStent, *n*	10
Age, years (average, range)	64 (23–92)
Gender, male/female	35/15
ASA status, *n* (%)	
Grade I	3 (6)
Grade II	11 (22)
Grade III	32 (64)
Grade IV	3 (6)
Grade unknown	1 (2)
**Procedures with consecutive Leakage (*n* = 40)**	
Surgical esophageal resection, *n*	15
Iatrogenic instrumental perforations, *n*	9
Bariatric gastric sleeve, *n*	6
Hiatal hernia repair, *n*	3
Boerhaave syndrome, *n*	2
Surgical resection of gastric cancer, *n*	2
Lung resection	1
LINX band explantation	1
Ingestion foreign body	1

Out of the 40 patients included in the study, the diagnosis of upper GI leak was confirmed in all cases. The leaks were either postoperative at an anastomosis or were iatrogenic and caused by an endoscopic or surgical procedure. Additionally, 10 patients who underwent resection of the tumor-bearing esophagus were treated with VACStent intraoperatively in a preemptive manner. These patients had significant prior diseases and neodjuvant chemo or radiochemo therapies.

The correct placement, positioning, and expansion of the VACStent was technically successful in all cases. The leaks as well as the anastomoses were completely covered and continuous suction with a mean negative suction pressure of −85 mmHg (range −75 to −125 mmHg) was installed in all cases. The mean indwelling time per VACStent was 5.2 days (range 2–8), and the mean indwelling time per patient (including multiple stenting) was 9.6 days (range 3–55) (Table 2). In one patient who underwent resection of the esophagus due to cancer, a small esophageal-tracheal fistula developed at a very high intrathoracic anastomosis (20 cm from the incisors). After consecutive treatment with 9 VACStents over 55 days the patient was clinically stable and able to swallow liquid and mashed food during the treatment. However, the fistula did not heal completely and was finally closed by surgery. Only one case of sponge loss was reported, which fully recovered, and no significant VACStent dislocations occurred. Minor local mucosa bleeding could be observed in three patients after withdrawal of the VACStent. Removal of the 92 VACStents was performed without major problems.

**Table 2:** VACStent treatment.

VACStent treatment (*n* = 92)	
VACStent number per patient AL, mean	2 (1–9)
VACStent lay time, days, mean	5.2 (2–8)
VACStent treatment time, mean	9.6 (3–55)
Complications	8/92 (8.7 %)
Mild mucosal bleeding after extraction	3
Stent migration	1
Sponge discontinuation	1
Application difficulty through plastic nose	1
Aspiration during extraction	2

Of the 40 patients with upper GI leak, 15 underwent resection in esophageal cancer, one was resected for lung cancer with infiltration of the esophagus. Nine patients had suffered iatrogenic perforation and six patients underwent sleeve gastrectomy. One patient had a LINX band invasion which was removed surgically, and the transmural gap was closed with the VACStent intraoperatively. Eight patients suffered from other diseases (Table 1).

Patients were treated with an average of two stents (1–9), total morphological healing of the leak was observed in 28 of 40 patients (70%) after VACStent treatment. In four patients the leak healed lateron after further treatments (surgery, SEMS) (Table 3).

**Table 3:** Outcome.

Outcome	
Persistent leak after VACStent treatment	12/40
Healing after further treatments	4
No healing	8
Complete closure	32/40 (80%)
Postoperative morbidity-AL rate, *n*	1/10
Sepsis progression, *n*	1/50
Death due to tumor situation, *n*	3/50

Preceded endoscopic therapies of the esophagus leakages were 24 implantations of an EsoSponge, 4 cSEMS, 1 OTSC, 1 OverStitch, 4 introductions of EVT with foil drainage fixed at feeding tube, 1 endoscopic wound rinsing. Multiple procedures per patient were possible. 9 patients had no endoscopic pretreatment (Table 4).

**Table 4:** Endoscopic treatments prior VACStent.

Previous endoscopic procedures, (*n* = 45, multiple procedures per patient)	
None	9
Esosponge	24
cSEMS	4
Open-pore film fixed to feading tube	4
OverStitch	1
OTSC	1
Balloon dilatation	1
Endoscopic wound rinsing	1

In the 10 patients who received preemptive VACStent treatment, all 12 VACStent applications were technically successful in all interventions. Patients were treated with a single VACStent, which was placed intraoperatively at the anastomosis. One patient developed esophageal leakage 10 days after the procedure, which was successfully treated with two additional VACStent applications for 14 days. All patients showed complete morphological healing of the anastomosis (100%). The median hospital stay was 16 days (range 11–29) and the ICU stay was 5 days (range 0–12).

In all cases except one, the VACStent treatment successfully controlled the septic focus of the leak, and there were no reported severe adverse events associated with the device (SADE). However, three deaths were recorded due to the patients’ tumor situation.

All patients had at least one follow-up visit between 2 weeks and 12 months after surgery, median follow-up time was 9 weeks. One patient had a stenosis 30 days after surgery with fully recovery at follow-up endoscopy 6 months after surgery. One patient suffered from dysphagia two weeks after hospital discharge. 9 out of 15 patients from the prospective study received follow-up endoscopy 12 months after surgery. One had a tumor relaps, one had an ulcus at the former anastomosis, and one patient continued to have a residual cavity.

## Discussion

So far, only very limited data from small retrospective and prospective patient series are available, since the VACStent is a new innovative medical device and the indication of esophageal application is rare (6–8). Especially with regard to technical applicability and safety, it is therefore highly desirable to create a broader data basis for these parameters.

This was the task of this summary analysis of three different prospective patient cohorts and was able to compile and evaluate the results of 92 VACStent applications for these target parameters, including the data from the already published prospective VACStent trial (8). In particular, we aimed to verify whether the previous conclusions from the VACStent trial are also confirmed in this broader pooled analysis.

The prospective register study and clinical trial both showed that the VACStent is effective in combining the benefits of stent therapy with successful endoscopic vacuum therapy for upper gastrointestinal tract leaks, anastomotic suture line failures, and as a preventive measure. Morphological healing was observed in 70% of patients after VACStent placement, which is comparable to healing rates expected with EVT through a PU sponge (10). With complementary therapies, the healing rate could be increased to 80%. The rate of healing is influenced by factors such as the chronicity and location of the leak or fistula and the level of suction vacuum applied (11). Despite the high heterogeneity and initial complicated conditions among the three patient cohorts, the healing rate was very satisfactory.

Endoscopic pretreatments were diverse and varied, suggesting that no established standard for leak closure has yet been established (12). Pure direct closure by suture (OverStitch), clip/OTSC or cSMES can be successful in fresh leaks without a relevant wound cavity, although the lack of drainage function is limiting for success. EVT procedures show the best results, with primary intracavitary sponge application accelerating wound clearance. However, the success of intraluminal sponge application corroborated also by the results of the VACStent show that suction promotes and enables healing of wound cavities even without direct wound contact.

Implantation, insertion and release of the VACStent was mostly rated as easy and moderately difficult in rare cases, and there was no significant difference from SEMS applications. Continuous negative pressure via the PU-sponge cylinder was simple and effective throughout the application period and dislocation during VACStent implantation was extremely rare. Furthermore, no significant migration was observed during the later course of treatment. The free passage through the VACStent body, which is a major advantage of the VACStent design principle, was mostly possible.

This study did not report any severe VACStent-associated complications (SADE reports) in any of the 92 VACStent applications. However, local moderate erosions or ulcers were reported in some cases in the area of the stent beads. These erosions or ulcers did not result in perforation or bleeding and did not require intervention.

Esophageal stenosis with clinical dysphagia is a potential complication of long-term sequela of endoluminal EVT and possibly VACStent therapy (8). During follow-up, the patients were visited and control endoscopies performed until 12 months post-surgery.

The outcomes of the three study cohorts demonstrate that the VACStent can be used for any leaks that can be covered with the sponge cylinder. It is recommended to use the VACStent as early as possible, preferably at the time of diagnosis, to prevent the formation of larger wound cavities and chronic fistulas. It is effective for leaks such as iatrogenic injuries (e.g., during ERCP, TEE), anastomotic suture line failures after esophageal resections, in bariatric surgery, or even in spontaneous esophageal rupture (Boerhaave syndrome). However, large wound cavities might be a limiting factor, as long as not a broad connection to the esophagus exists.

Limitation of this study is the restricted sample size due to the initial low availability of the VACStent. Further studies with larger numbers of cases are necessary and are being planned.

## Conclusion

Based on the results of the study, the VACStent appears to be a safe and effective treatment option for upper GI tract leaks and anastomotic suture line failures. The VACStent combines the advantages of EVT with those of stenting, allowing for immediate wound closure and effective drainage of endoluminal wound cavities. The VACStent also controls the septic focus, promotes accelerated wound healing, and provides an open passage for rapid postoperative feeding and endoscopic access distal to the leak. Overall, the VACStent has the potential to improve clinical outcomes in resections and perforations of difficult to treat esophageal-gastric anastomoses in oncological and bariatric surgery.

## Data Availability

The raw data supporting the conclusions of this article will be made available by the authors, without undue reservation.
